# Inpatient Outcomes of Pulmonary Embolism in Patients with Inflammatory Bowel Disease: Insights from a Nationwide Analysis

**DOI:** 10.3390/jcm15145328

**Published:** 2026-07-08

**Authors:** Uday Sankar Akash Vankayala, Chloe Lahoud, Bivin George, Ali Sohail, Bahy Abofrekha, John Afif, Omar Abureesh, Suzanne El-Sayegh, Hassan Al Moussawi

**Affiliations:** 1 Department of Internal Medicine, Staten Island University Hospital, Northwell Health, 475 Seaview Avenue, Staten Island, NY 10305, USA; 2 Department of Internal Medicine, Mount Sinai West, New York, NY 10019, USA; 3 Division of Gastroenterology, Department of Internal Medicine, Staten Island University Hospital, Northwell Health, 475 Seaview Avenue, Staten Island, NY 10305, USA

**Keywords:** inflammatory bowel disease, pulmonary embolism, inpatient outcomes, in-hospital mortality, resource utilization

## Abstract

**Background:** Inflammatory bowel disease (IBD) is a chronic inflammatory disorder that confers an increased risk of venous thromboembolism (VTE) and subsequent pulmonary embolism (PE). The risk stems from chronic systemic inflammation promoting endothelial dysfunction and hypercoagulability. Data on specific inpatient outcomes and procedural needs in patients with IBD with acute PE remains limited. This study explores these outcomes at a national level. **Methods:** We conducted a Nationwide Inpatient Sample (NIS) database analysis (2016–2020). Adult hospitalizations for acute PE were identified using ICD-10-CM codes and stratified based on IBD status. Multivariable regression analysis was performed to determine independent associations between IBD status and in-hospital mortality, length of stay (LOS), cardiac complications, and ICU-level interventions (intubation, central venous catheterization (CVC), arterial line placement, requirement of vasopressors), and blood transfusion. **Results:** Among 377,143 acute PE hospitalizations, 4123 (1.1%) had IBD. Patients with IBD were younger (58.72 vs. 62.78 years, *p* < 0.001) and had lower prevalence of diabetes mellitus, hypertension, end-stage renal disease (ESRD), dyslipidemia, overweight/obesity, coronary artery disease and smoking status (*p* < 0.05). Despite a favorable baseline profile, patients with IBD had a longer length of stay (LOS) (8.82 vs. 7.30 days, *p* < 0.001) but no significant association with in-hospital mortality (aOR = 0.93, *p* = 0.281). Multivariable analysis showed patients with IBD had higher odds of requiring CVC placement (OR = 1.42, *p* < 0.001), vasopressors (OR = 1.22, *p* = 0.05), and blood transfusions (OR = 1.78, *p* < 0.001). Conversely, they had lower odds of cardiac arrest (OR = 0.64, *p* < 0.001) and cor pulmonale (OR = 0.32, *p* = 0.012). **Conclusions:** patients with IBD with acute PE represent a complex population with high resource utilization. Future research is needed the development of IBD-specific PE risk stratification, targeted management, prophylactic and therapeutic anticoagulation guidelines.

## 1. Introduction

Inflammatory bowel disease (IBD), comprising Crohn’s disease (CD) and ulcerative Colitis (UC), is a chronic inflammatory disorder of the gastrointestinal (GI) tract affecting 2.4 to 3.1 million individuals in the United States [1]. Involvement of systems outside the GI tract is termed extraintestinal manifestations (EIMs), which occurs in approximately 25% of patients with IBD [2,3,4]. Venous thromboembolism (VTE) is a significant EIM with potentially life-threatening complication. IBD confers a two- to three-fold higher risk of VTE compared to individuals without IBD stemming from a complex interplay of the coagulation cascade and immune system with pulmonary embolism (PE) occurring at an annual incidence of 0.2% [5,6].

The heightened VTE risk in IBD reflects multiple pathophysiological mechanisms satisfying Virchow’s triad. Chronic systemic inflammation in IBD creates a prothrombotic state characterized by both arterial and venous vascular complications [7]. Inflammatory cytokines (TNF-α, IL-6, IL-1β) induce endothelial dysfunction with upregulation of tissue factor and downregulation of natural anticoagulants (protein C, protein S, antithrombin). Platelet activation via the CD40/CD40L axis amplifies both inflammatory and thrombotic cascades. The coagulation system is further dysregulated through elevated pro-coagulant factors (fibrinogen, factor V, factor VIII, von Willebrand factor) and impaired fibrinolysis from increased plasminogen activator inhibitor-1 [8,9,10,11]. Recent insights demonstrate layilin downregulation in IBD, which inhibits integrin activation, thereby promoting platelet hyperreactivity [12]. Additionally, intestinal dysbiosis, bacterial translocation, active disease flares, immobilization, corticosteroid therapy, and IBD-related surgery collectively heighten thrombotic risk, particularly during disease flares [13,14,15].

Despite well-established epidemiology on VTE incidence in IBD; data on inpatient outcomes such as mortality, cardiopulmonary complications, intensive care and resource utilization in patients with IBD who develop PE remain limited [16,17]. Current PE risk stratification tools derived from general populations may not adequately capture inflammatory and treatment-related factors unique to IBD. Our nationwide study aims to address this gap and comparing in-hospital mortality, length of stay, cardiopulmonary complications, and resource utilization profiles between patients with and without IBD hospitalized with acute PE.

## 2. Materials and Methods

### 2.1. Study Design and Data Source

This study utilized a retrospective cohort design from January 2016 to December 2020 utilizing the Nationwide Inpatient Sample (NIS). We identified adults aged 18 years and older hospitalized with a primary diagnosis of acute pulmonary embolism (PE) via International Classification of Diseases, Tenth Revision, Clinical Modification (ICD-10-CM) codes (please refer to Appendix A Table A1). Patients with prior deep vein thrombosis (DVT) or PE were excluded to capture incident events. The final cohort was stratified by the presence or absence of inflammatory bowel disease (Crohn’s disease and ulcerative colitis).

### 2.2. Baseline Characteristics and Comorbidities

Baseline demographic variables including age, sex, race/ethnicity, primary insurance payer, and income were extracted, along with comorbid conditions such as diabetes mellitus, hypertension, chronic kidney disease, end-stage renal disease (ESRD), dyslipidemia, overweight/obesity, coronary artery disease, and smoking status. Overall comorbidity burden was assessed using standardized NIS comorbidity measures.

### 2.3. Outcomes

The exposure of interest was IBD status. The primary outcome of interest were in-hospital mortality and length of stay (LOS). Secondary outcomes included cardiopulmonary complications (acute right heart failure, cor pulmonale, chronic thromboembolic pulmonary hypertension (CTEPH), and cardiac arrest) and markers of inpatient acuity/resource utilization (arterial line placement, central venous catheter placement, vasopressor use, and endotracheal intubation, and blood transfusion) during hospitalization.

### 2.4. Statistical Analysis

Qualitative data described as number and percentage and compared using chi-square test and Fisher’s exact test. Multivariable binary logistic regression was performed to assess whether IBD was independently associated with the outcomes, adjusting for multiple covariates and demographic variables (age, sex, race, DM, HTN, dyslipidemia, overweight, obesity, CKD stage 3–5, ESRD, smoking and CAD). Results are presented as odds ratio (OR) estimates with 95% confidence intervals (CIs). The level of significance was taken at *p*-value ≤ 0.05. All statistical analyses were conducted using IBM Statistical Package for Social Sciences (SPSS) Statistics Software Version 30.0, IBM Corp., Chicago, IL, USA.

## 3. Results

### 3.1. Patient Demographics

Our analysis yielded a total of 377,143 hospitalizations for acute PE. Among these, *n* = 4123 (1.1%) patients had coexistent IBD, while *n* = 373,020 did not. Patients with IBD were younger than those without IBD (58.7 ± 16.6 vs. 62.8 ± 16.9 years, *p* < 0.001) and more often female (54% vs. 51.1%, *p* < 0.001) and White (81.8% vs. 70.5%, *p* < 0.001). The IBD cohort had a lower prevalence of diabetes mellitus (18.4% vs. 25%), hypertension (49.6% vs. 61.3%), obesity (18.5% vs. 23.6%), dyslipidemia, chronic kidney disease stage 3–5, end-stage renal disease, and coronary artery disease (all *p* < 0.01). However, smoking prevalence was higher in the IBD cohort (27% vs. 24.8% *p* = 0.002). Socioeconomic analysis showed that patients with IBD were more likely to hold private insurance (34.2% vs. 26.3%) and reside in the highest income quartiles (*p* < 0.001).

Demographic and clinical data are summarized in Table 1.

### 3.2. Primary Outcomes

Patients with IBD compared to those without IBD had lower unadjusted odds of in-hospital mortality (5.8% vs. 7.0%, *p* = 0.004). However, after adjustment, IBD was not independently associated with in-hospital mortality (aOR 0.929, 95% CI 0.81–1.06, *p* = 0.281). Increasing age was strongly associated with mortality (OR 1.023 per year, *p* < 0.001), whereas female sex was associated with a reduced risk of death (OR 0.875, *p* < 0.001). Compared to whites; Asians faced the highest mortality risk followed by Black, Hispanic, Native American, and other race groups (all *p* < 0.001). Among comorbidities, end-stage renal disease (ESRD) was the strongest predictor of mortality, with nearly threefold risk.

Multivariable regression analysis for in-hospital mortality summarized in Table 2.

An ANOVA analysis of continuous variable of LOS revealed patients with IBD with PE experienced longer LOS than patients without IBD (8.82 ± 11.74 vs. 7.30 ± 10.15 days, *p* < 0.001).

### 3.3. Secondary Outcomes

#### 3.3.1. ICU Level Interventions and Resource Utilization

Patients with IBD demonstrated a distinct resource utilization profile, characterized by lower odds of intubation (OR 0.856, *p* = 0.010) but significantly higher odds of central venous catheterization (OR 1.426, *p* < 0.001), need for blood transfusion (OR 1.78, *p* < 0.001) and vasopressor use (OR 1.22, *p* = 0.05). In contrast, no significant difference was noted in arterial line utilization (OR 1.05, *p* = 0.74).

Multivariable regression analysis of ICU Level interventions and resource utilization profile summarized in Table 3. Detailed analysis listed in Appendix A Table A2.

#### 3.3.2. Cardiac Complications

Patients with IBD showed significantly lower odds of specific cardiac events. Lower risk of in-hospital cardiac arrest (OR 0.65, *p* < 0.001) and cor pulmonale (OR 0.32, *p* = 0.012). A lower trend towards acute right heart failure was observed (OR 0.496, *p* = 0.065), but no significant association between IBD status and Chronic Thromboembolic Pulmonary Hypertension (CTEPH). Traditional risk factors, particularly obesity, CKD, and ESRD, remained the primary drivers of right-sided heart complications across the entire cohort.

Multivariate regression analysis of Cardiac complications summarized in Table 4 and Detailed analysis with adjustments in Appendix A Table A3.

**Figure 1:**
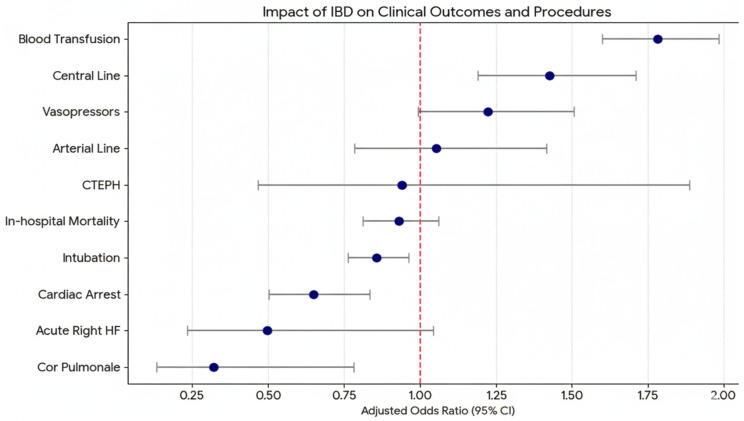
Adjusted odds ratios for clinical outcomes and procedural interventions in patients with IBD. A forest plot illustrating the independent association of inflammatory bowel disease (IBD) with various inpatient outcomes and procedures. Odds ratios (OR) were determined via multivariate logistic regression, adjusting for age, sex, race, and clinical comorbidities (DM, HTN, obesity, CKD, and CAD). The red dashed line represents the null effect (OR = 1.0). Error bars denote 95% confidence intervals (CI).

A summary of the adjusted odds ratio for clinical outcomes and procedural interventions in patients with IBD given below in Figure 1:

## 4. Discussion

This nationwide analysis of 377,143 adult acute pulmonary embolism (PE) hospitalizations identified 4123 (1.1%) patients with comorbid inflammatory bowel disease (IBD). Patients with IBD hospitalized with acute PE were significantly younger (58.72 vs. 62.78 years, *p* < 0.001), more frequently female (54% vs. 51.1%, *p* < 0.001) with a lower prevalence of DM, HTN, obesity, dyslipidemia, CKD stage 3–5, ESRD, and CAD (all *p* < 0.01). Paradoxically, patients with IBD experienced a longer hospital duration compared to the non-IBD cohort (8.82 ± 11.74 vs. 7.30 ± 10.15 days, *p* < 0.001). The primary outcomes of mortality after multivariate adjustment was not associated with IBD status (aOR 0.929, 95% CI 0.81–1.06, *p* = 0.281). Patients with IBD demonstrated higher odds of central venous catheterization (OR 1.426, *p* < 0.001), blood transfusion (OR 1.78, *p* < 0.001), borderline increased vasopressor use (OR 1.22, *p* = 0.05), and lower odds of intubation (OR 0.856, *p* = 0.010). Conversely, they had lower odds of cardiopulmonary complications including in-hospital cardiac arrest (OR 0.65, *p* < 0.001), cor pulmonale (OR 0.32, *p* = 0.012) and acute right heart failure (OR 0.496, *p* = 0.065). Collectively these findings suggest IBD is an independent risk factor PE, yet the severity of PE-related complications appears to be dictated by the burden of traditional cardiovascular risk factors rather than IBD-specific inflammation.

The younger age of the IBD cohort is consistent with prior reports by Aldiabat et al. [18] and Kappelman et al. [19], who demonstrated a twofold increase in PE incidence among younger patients with IBD, even after age- and sex-matched analyses. This paradox reflects the intrinsic prothrombotic milieu of IBD driven by mechanisms independent of traditional pathways. IBD creates a unique hypercoagulable state through multiple acquired abnormalities including elevated levels of pro-coagulant factors (fibrinogen, factor V, factor VIII, von Willebrand factor), acquired protein C resistance independent of Factor V Leiden mutation, elevated homocysteine levels due to malabsorption of folate and vitamin B12, increased platelet count and reactivity during active inflammation, and impaired fibrinolysis from elevated plasminogen activator inhibitor-1 [7].

At the molecular level, increased expression of CD40 and CD40L in the gut mucosa, with release of CD40L by activated platelets, generates a robust pro-inflammatory and pro-thrombotic response [16]. This leads to downstream activation of atherosclerosis and thrombosis pathways, specifically amplified during disease flares by elevation of acute-phase reactants including CRP, interleukin-6 (IL-6), tumor necrosis factor-alpha (TNF-α) [10,11,20,21]. IBD-specific genetic variants (NOD2, ATG16L1, IL23R) and classic coagulation polymorphisms (MTHFR, PAI-1) impair bacterial clearance and induce prolonged inflammatory and prothrombotic states [22,23]. Recent insights on layilin downregulation in patients with IBD permits unopposed integrin-mediated platelet aggregation, suggesting distinct thrombogenic mechanisms producing structurally and compositionally different clots [24]. Corticosteroid therapy enhances platelet aggregation, increases synthesis of clotting factors, and reduces anticoagulant protein levels [25]. Prolonged immobilization and IBD-related surgical procedures create high-risk perioperative states contributing to venous stasis and hypercoagulability [24,25]. This highlights the need for increased surveillance across all age groups in IBD, particularly during active disease flares.

Despite this hypercoagulable state, IBD was not associated with in-hospital mortality after multivariable adjustment (aOR 0.929, *p* = 0.281). This paradox can be explained by converging mechanisms. First, the younger age of the IBD cohort compared to the non-IBD cohort (58.72 vs. 62.78 years, *p* < 0.001) provides superior cardiopulmonary reserve to tolerate acute PE-induced hemodynamic stress. Less age-related myocardial degeneration and better Frank–Starling recruitment enable enhanced compensatory mechanisms [26,27,28]. Second, the lower prevalence of the strongest mortality predictors ESRD (OR 2.976 for mortality), DM and CKD stage 3–5 spares the younger IBD population from comorbidity-specific complications such as volume overload exacerbating RV strain, electrolyte abnormalities triggering arrhythmias. Additionally, the lower prevalence of dyslipidemia in the IBD cohort (19.2% vs. 24.9%, *p* < 0.001) may translate to differential statin exposure, which independently reduces 30-day PE mortality through pleiotropic anti-inflammatory mechanisms in the RIETE registry [29]. Third, early detection bias through closer medical surveillance and imaging may incidentally detect PE with lower clot burdens in minimally symptomatic or asymptomatic patients [5,15,30]. Heightened clinician awareness in this population lowers the VTE diagnostic threshold [5,30]. The demographic concentration of the IBD cohort in the metropolitan areas (>73% in populations >250,000) with private insurance (34.2% vs. 26.3%, *p* < 0.001) ensures superior access to multidisciplinary management supporting early detection [31,32]. This in turn may manifest as lower rates of cardiac arrest (OR 0.65, *p* < 0.001), cor pulmonale (OR 0.32, *p* = 0.012), and intubation (OR 0.856, *p* = 0.010).

Moreover, clot physiology in IBD may differ, with more friable, platelet-rich with neutrophil extracellular traps (NETs) that fragment into smaller peripheral emboli rather than evolving into massive central obstructions [33,34]. Additionally, medication exposure to anti-inflammatory therapies biologics and immunosuppressants may reduce massive PE incidence [9,16]. These findings of equivalent mortality in the IBD align with the observations by Dorn et al. [35] and Fumery et al. [36], who demonstrated no increased risk of cardiovascular mortality or stroke attributable to IBD itself.

Notably, multivariable analysis of the in-hospital mortality showed protective associations of hypertension (OR 0.73, *p* < 0.001), obesity (OR 0.766, *p* < 0.001), dyslipidemia (OR 0.876, *p* < 0.001), and smoking (OR 0.756, *p* < 0.001). These findings mirror the “obesity paradox” in acute PE, where patients with higher baseline thrombotic risks demonstrate lower short-term mortality. Obese patients may experience more pronounced dyspnea leading to earlier symptom recognition, imaging, and treatment reducing the severity at presentation. Adiposity may confer metabolic and neurohumoral protective effects, including higher circulating levels of cardioprotective adipokines and enhanced ischemic preconditioning [37]. Finally, obese patients are more likely to receive baseline thromboprophylaxis [38]. Alternatively, patients with massive PE causing immediate hemodynamic collapse may not survive long enough for comorbidities to be documented, creating survivor bias that produces the appearance of protective effects for chronic conditions [31,32].

Patients with IBD experienced significantly longer lengths of stay compared to patients without IBD (8.82 ± 11.74 vs. 7.30 ± 10.15 days, *p* < 0.001), consistent with findings by Wang et al. [39], who demonstrated an average 8-day longer stay hospitalization. This prolonged LOS reflects IBD-specific clinical vulnerabilities prolonging the LOS. Infection susceptibility secondary to immunocompromised state, disease flares, procedural related complications, nutritional deficiencies, surgical procedures and recovery together prolong LOS [25]. Corticosteroids and Clostridium difficile are the most established drivers of LOS and morbidity [24,40,41]. The need for coordinated multidisciplinary management also prolongs hospitalization [39,42].

Patients with IBD demonstrated higher odds of central venous catheterization (CVC; OR 1.426, *p* < 0.001), borderline increased vasopressor use (OR 1.223, *p* = 0.05) yet significantly lower odds of intubation (OR 0.856, *p* = 0.010), yet no difference in arterial line utilization (OR 1.053, *p* = 0.735). The increase in vasopressor use can be explained by inflammation-mediated vasodilation through induction of nitric oxide synthase expression in vascular smooth muscle, causing arteriolar vasodilation and reduced systemic vascular resistance. Capillary leak from cytokine-mediated barrier dysfunction causes intravascular volume depletion, necessitating vasopressor support when fluid resuscitation alone remains insufficient. Relative adrenal insufficiency in patients on chronic corticosteroid therapy impairs catecholamine responsiveness during acute stress, causing refractory hypotension that may require pressor support and CVC access [43,44,45]. The higher CVC utilization likely reflects both hemodynamic management needs and IBD-specific clinical requirements such as poor peripheral venous access, prolonged intravenous treatment with corticosteroids, biologics, or total parenteral nutrition (TPN) [46].

Patients with IBD were nearly 1.8 times more likely to require blood transfusions than individuals without IBD (OR 1.783, *p* < 0.001). Anemia in IBD is multifactorial, driven by several mechanisms that result in low baseline hemoglobin levels and lower the threshold for transfusion: impaired iron absorption from duodenal and jejunal inflammation; IL-6-driven hepcidin upregulation, which blocks intestinal iron absorption and traps iron within macrophage stores; vitamin B12 deficiency from terminal ileal disease or surgical resection; folate malabsorption compounded by sulfasalazine and methotrexate use; medication-induced myelosuppression from thiopurines (azathioprine, 6-mercaptopurine) [46,47,48,49]. Given the hemodynamic stress from PE and anticoagulation, clinician judgment plays a determinant role in the maintenance of higher baseline threshold for transfusions. Future studies incorporating baseline hemoglobin levels and transfusion triggers are needed to distinguish truly increased transfusion requirements from practice variation.

Conversely, patients with IBD demonstrated lower odds of intubation (OR 0.856, *p* = 0.010) despite higher vasopressor and CVC requirements. Intubation in acute PE is primarily necessitated by massive PE causing obstructive cardiogenic shock, cardiac arrest, or refractory hypoxemia [27,28]. Earlier detection through heightened clinical surveillance and lower diagnostic thresholds likely identifies PE with smaller, more peripheral clot burdens insufficient to cause acute respiratory failure [5,30]. Additionally, the younger age of the IBD cohort provides greater respiratory reserve, enabling compensation for moderate hypoxemia without requiring mechanical ventilation. The absence of increased arterial line use (OR 1.053, *p* = 0.735) further supports transient hemodynamic compromise being responsive to initial interventions rather than progressive shock. Collectively, these findings suggest that patients with IBD experience hemodynamic instability from inflammation-mediated vasodilation rather than respiratory failure from massive PE, representing a fundamentally different clinical trajectory.

This distinct pattern of ICU-level resource utilization extends to the cardiac complications profile, where patients with IBD similarly demonstrated lower odds of severe PE-related cardiac events.

On adjusted multivariable analysis, patients with IBD had lower odds of cor pulmonale (OR 0.324, *p* = 0.012) and in-hospital cardiac arrest (OR 0.647, *p* < 0.001). No significant association with CTEPH (OR 0.940, *p* = 0.862), acute right heart failure (OR 0.496, *p* = 0.065). These complications result from acute RV pressure overload caused by massive proximal clot burden, leading to RV dilation, septal bowing, and obstructive cardiogenic shock. The lower odds in the IBD cohort, paralleling lower intubation rates, suggest presentation with lower-severity PE events reflecting earlier detection at smaller clot burdens. The persistence of lower odds despite adjustment indicates that established cardiovascular risk factors drive PE-related cardiac sequelae rather than IBD-specific inflammation, consistent with Dorn et al. [35] and Fumery et al. [36], who demonstrated no increased risk of cardiovascular mortality or stroke attributable to IBD. While IBD-associated inflammation promotes acute thrombosis through the mechanisms described above, it does not appear to impair thrombus resolution or drive maladaptive pulmonary vascular remodeling, as evidenced by the absence of association with CTEPH. This dissociation between acute thrombotic risk and long-term cardiac sequelae warrants further investigation through longitudinal studies, as our inpatient dataset cannot adequately assess outcomes such as CTEPH development.

## 5. Conclusions

In conclusion, patients with IBD with acute PE represent a unique phenotype characterized by younger age, lower comorbid burden, yet associated with greater resource utilization and longer hospital lengths of stay. While multivariable adjustment reveals paradoxically lower rates of cardiac arrest, cor pulmonale and intubations. This establishes that IBD independently increases the risk of PE but the acute hemodynamic and cardiac consequences are still primarily determined by traditional comorbidities. It suggests that IBD-associated PE follows a different clinical trajectory that needs to be further studied and managed optimally considering the disease-specific risk factors. Clinicians must maintain a high index of suspicion for PE in this population and implement proactive and multidisciplinary care to mitigate hospital resource burden.

Future research should focus on understanding links between IBD inflammation and thrombotic complications, developing IBD-specific PE risk stratifications, individualized treatments and developing anticoagulation guidelines.

## 6. Limitations

This study has important limitations that needs to be taken into consideration. One of the most important limitations is that the NIS database lacks PE severity assessment tools essential for risk stratification and outcome interpretation. We could not evaluate right ventricular dysfunction, which is the cornerstone of PE severity classification. Similarly, biomarker data for risk stratification remain unavailable, such as cardiac troponin elevation, brain natriuretic peptide or N-terminal pro-BNP, and lactate levels. Clot burden and anatomic location cannot be assessed to determine the hemodynamic and severity impact. We could not differentiate massive central PE from sub-massive PE or low-risk PE. Additionally, validated prognostic scores Pulmonary Embolism Severity Index (PESI) and simplified PESI cannot be calculated, as triage data is unavailable. This prevents determination of whether the IBD and non-IBD cohorts had equivalent PE severity or whether observed outcome differences reflect differential disease severity driven by early detection bias versus genuinely different pathophysiology.

Furthermore, the NIS database as discussed lacks medication exposure data preventing assessment of how pharmacologic therapies influence outcomes. IBD-specific therapies that reduce the inflammatory burden, including biologic agents (anti-TNF, anti-IL-12/23, anti-integrin), immunosuppressants (azathioprine, methotrexate), corticosteroids and anticoagulation treatments could not be evaluated. Additionally, IBD activity status at PE presentation, inflammatory biomarker levels (C-reactive protein, fecal calprotectin), and IBD phenotype could not be assessed, limiting mechanistic insights into inflammation-thrombosis relationships.

The observational study design limits causal inference despite multivariable adjustment for confounders. Our analysis is restricted to the inpatient setting and cannot evaluate long-term outcomes including recurrent VTE rates, chronic thromboembolic pulmonary hypertension development (typically diagnosed 3–6 months post-PE), post-PE syndrome, or quality of life. The NIS database structure lacks unique patient identifiers enabling longitudinal tracking across hospitalizations or calendar years. While we excluded patients with documented VTE history during the index hospitalization, we could not definitively exclude patients with VTE events in prior admissions occurring in different years or facilities, potentially including some recurrent events. However, recurrent VTE represents a minority of cases (~15–20%), and this limitation affects both cohorts equally.

Additionally, patients with IBD may undergo more frequent diagnostic imaging for IBD assessment, creating detection bias where incidental subsegmental PE is identified in asymptomatic patients, inflating PE incidence while improving apparent outcomes through identification of lower-severity cases. Finally, coding inaccuracies inherent to administrative databases may introduce misclassification bias, though the large sample size (377,143 PE admissions) and use of validated ICD-10-CM algorithms likely mitigate this concern.

## Figures and Tables

**Table 1:** Baseline characteristics and demographics of patients.

Variable	IBD (*n* = 4123)	No IBD (*n* = 373,020)	*p*-Value
Age (mean ± SD)	58.72 ± 16.57	62.78 ± 16.85	<0.001
Sex female (mean)	2225 (54%)	190,458 (51.1%)	<0.001
Race			<0.001
White	3274 (81.8%)	254,804 (70.5%)	
Black	466 (11.6%)	66,100 (18.3%)	
Hispanic	141 (3.5%)	25,068 (6.9%)	
Asian	25 (0.6%)	4962 (1.4%)	
Native American	19 (0.5%)	1745 (0.5%)	
Other	77 (1.9%)	8924 (2.5%)	
Primary expected payer			<0.001
Medicare	1988 (48.3%)	195,150 (52.4%)	
Medicaid	497 (12.1%)	52,648 (14.1%)	
Private	1407 (34.2%)	97,995 (26.3%)	
Self-pay	115 (2.8%)	14,988 (4%)	
None	9 (0.2%)	1312 (0.4%)	
Other	104 (2.5%)	10,364 (2.8%)	
Population Setting			0.008
>1 million in central city	1111 (27%)	104,401 (28.1%)	
>1 million on fringes of city	1089 (26.5%)	91,002 (24.5%)	
250 K–1 million population	840 (20.4%)	76,138 (20.5%)	
50 K–250 K population	416 (10.1%)	36,527 (9.8%)	
<50 K metropolitan counties	407 (9.9%)	36,387 (9.8%)	
<50 K Not metropolitan counties	250 (6.1%)	26,747 (7.2%)	
Median Household Income			
0–25th percentile	974 (24%)	108,817 (28.7%)	<0.001
26–50th percentile	1030 (25.4%)	97,530 (26.6%)	<0.001
51–75th percentile	1063 (26.2%)	87,619 (23.9%)	<0.001
76–100th percentile	987 (24.3%)	72,383 (19.8%)	<0.001
Comorbidities			
DM	758 (18.4%)	93,379 (25%)	<0.001
HTN	2047 (49.6%)	228,599 (61.3%)	<0.001
Dyslipidemia	791 (19.2%)	92,885 (24.9%)	<0.001
Overweight BMI 25 to 29.9	132 (3.2%)	8699 (2.3%)	<0.001
Obesity BMI 30+	762 (18.5%)	87,954 (23.6%)	<0.001
Smoking	1111 (27%)	92,528 (24.8%)	0.002
CKD 3–5	286 (6.9%)	30,174 (8.1%)	0.007
ESRD	56 (1.4%)	7591 (2%)	0.002
CAD	541 (13.1%)	64,145 (17.2%)	<0.001

Baseline characteristics and demographics of patients hospitalized with acute pulmonary embolism, stratified by inflammatory bowel disease (IBD) status.

**Table 2:** Multivariate regression analysis for in-hospital mortality.

Variable	Adjusted OR	Low CI 95%	High CI 95%	*p*-Value
IBD	0.929	0.813	1.062	0.281
Age (per year)/mean	1.023	1.022	1.024	<0.001
Sex (female)	0.875	0.853	0.898	<0.001
Race Ref: White				
Black	1.162	1.122	1.203	<0.001
Hispanic	1.371	1.306	1.438	<0.001
Asian	1.832	1.678	2.00	<0.001
Comorbidities				
DM	1.24	1.203	1.279	<0.001
HTN	0.73	0.709	0.752	<0.001
Dyslipidemia	0.876	0.849	0.904	<0.001
Overweight: BMI 25 to 29.9	0.987	0.906	1.075	0.763
Obesity BMI 30+	0.766	0.739	0.793	<0.001
CKD 3–5	1.178	1.127	1.232	<0.001
ESRD	2.976	2.789	3.177	<0.001
Smoking	0.756	0.732	0.780	<0.001
CAD	0.944	0.912	0.978	0.001

Multivariate regression analysis for in-hospital mortality among patients hospitalized with acute pulmonary embolism (PE), stratified by inflammatory bowel disease (IBD) status.

**Table 3:** Multivariate regression analysis of ICU Level interventions and resource utilization profile in patients with IBD.

Outcomes	Adjusted OR	Lower CI 95%	Higher CI 95%	*p*-Value
Inflammatory Bowel Disease (IBD)				
Intubation	0.856	0.761	0.964	0.01
Arterial line	1.053	0.783	1.415	0.735
Central line	1.426	1.189	1.711	<0.001
Blood transfusion	1.783	1.602	1.986	<0.001
Vasopressors	1.223	0.992	1.507	0.05

ICU Level interventions and resource utilization profile in patients with IBD after multivariate regression analysis and post-adjustments for Age, Sex, Race, Comorbidities (DM, HTN, CKD, ESRD, Obesity, Dyslipidemia, Smoking, and CAD).

**Table 4:** Multivariate analysis of cardiac complications.

Outcomes	Adjusted OR	Lower CI 95%	Higher CI 95%	*p*-Value
Inflammatory Bowel Disease (IBD)				
Acute Right HF exacerbation	0.496	0.236	1.044	0.065
Cor pulmonale	0.324	0.135	0.781	0.012
Chronic thromboembolic pulmonary HTN	0.94	0.468	1.889	0.862
Cardiac arrest	0.647	0.501	0.834	<0.001

Multivariate analysis showing cardiac complications in pulmonary embolism patients with inflammatory bowel disease (IBD), adjusted for age, sex, race, comorbidities (DM, HTN, CKD, ESRD, obesity, dyslipidemia, smoking, CAD).

## Data Availability

The data that support the findings of this study are available from the Healthcare Cost and Utilization Project (HCUP) Nationwide Inpatient Sample (NIS). Data are available from the HCUP Central Distributor (https://www.hcup-us.ahrq.gov accessed on 1 January 2016) for researchers who meet the criteria for access, which includes completion of a Data Use Agreement training course and submission of an online Data Use Agreement. Statistical code and analysis protocols are available from the corresponding author upon reasonable request.

## Abbreviations

The following abbreviations were used in this manuscript:

aOR	Adjusted Odds Ratio
ANOVA	Analysis of Variance
BMI	Body Mass Index
CAD	Coronary Artery Disease
CD	Crohn’s Disease
CD40L	CD40 Ligand
CI	Confidence Interval
CKD	Chronic Kidney Disease
CRP	C-Reactive Protein
CTEPH	Chronic Thromboembolic Pulmonary Hypertension
CVC	Central Venous Catheter/Catheterization
DM	Diabetes Mellitus
DVT	Deep Vein Thrombosis
EIM	Extraintestinal Manifestation
ESRD	End-Stage Renal Disease
GI	Gastrointestinal
HF	Heart Failure
HTN	Hypertension
IBD	Inflammatory Bowel Disease
ICD-10-CM	International Classification of Diseases, Tenth Revision, Clinical Modification
ICU	Intensive Care Unit
IL-6	Interleukin-6
LOS	Length of Stay
NIS	Nationwide Inpatient Sample
OR	Odds Ratio
PE	Pulmonary Embolism
RV	Right Ventricular/Right Ventricle
SD	Standard Deviation
SPSS	Statistical Package for the Social Sciences
TNF-α	Tumor Necrosis Factor-Alpha
TPN	Total Parenteral Nutrition
UC	Ulcerative Colitis
VTE	Venous Thromboembolism

## Appendix A

**Table A1:** ICD-10 codes employed in the analysis.

IBD	K500, K501, K508, K509, K51
Acute pulmonary embolism (PE)	I26
Diabetes Mellitus (DM)	E08, E09, E10, E11, E13
Hypertension (HTN)	I10
Dyslipidemia (DLD)	E780, E781, E782, E783, E784, E785
Overweight BMI 25 to 29.9	Z6825, Z6826, Z6827, Z6828, Z6829, E663
Obesity BMI 30+	E6601, E6609, E661, E662, E668, E669, O99210, O99211, O99212, O99213, O99214, O99215, R939, Z6830, Z6831, Z6832, Z6833, Z6834, Z6835, Z6836, Z6837, Z6838, Z6839, Z6841, Z6842, Z6843, Z6844, Z6845, Z6854
Smoking	F17200, Z87891
CKD 3–5	N183, N184, N185
ESRD	N186
Coronary Artery Disease (CAD)	I2510, I252, I258, I259
Chronic DVT	I82.291, I82.211, I82.5, I82.7, I82.A2, I82.B2, I82.C2
History of PE	I2782
Acute right heart failure	I50811, I50813
Cor pulmonale	I2781
Chronic thromboembolic pulmonary hypertension	I2724
Cardiac Arrest	I462, I468, I469
Intubation	0BH17EZ, 0BH18EZ, 5A1935Z, 5A1945Z, 5A1955Z
A line	4A133B1
Central line	06HN33Z, 06HM33Z, 05H633Z, 05H533Z, 05HN33Z, 05HM33Z, 05HM3DZ
Vasopressor	3E033XZ, 3E043XZ
Blood transfusion	30233N1

**Table A2:** Detailed multivariable regression analysis of ICU Level interventions and resource utilization profile in patients with IBD.

Variable	Intubation	Arterial Line	Central Line	Vasopressors	Blood Transfusions
OR (95% CI); *p*-value					
IBD	0.856 (0.76–0.96); 0.010	1.053 (0.78–1.42); 0.735	1.426 (1.19–1.71); <0.001	1.223 (0.99–1.51); 0.050	1.783 (1.60–1.99); <0.001
Age (mean)	0.999 (0.99–0.99); <0.001	0.994 (0.99–0.99); <0.001	0.994 (0.99–0.99); <0.001	1.003 (1.00–1.00); 0.001	0.999 (0.99–1.00); <0.022
Sex (Female)	0.750 (0.73–0.77); <0.001	0.755 (0.71–0.81); <0.001	0.945 (0.90–0.99); 0.012	0.817 (0.78–0.86); <0.001	1.363 (1.32–1.40); <0.001
Race Ref: White					
Black	1.178 (1.14–1.21); <0.001	1.223 (1.13–1.33); <0.001	1.188 (1.12–1.26); <0.001	1.178 (1.11–1.25); <0.001	1.425 (1.38–1.48); <0.001
Hispanic	1.329 (1.28–1.39); <0.001	1.308 (1.17–1.47); <0.001	1.376 (1.27–1.49); <0.001	1.337 (1.23–1.46); <0.001	1.466 (1.39–1.54); <0.001
Asian	1.504 (1.38–1.64); <0.001	1.574 (1.26–1.97); <0.001	1.530 (1.31–1.79); <0.001	1.686 (1.44–1.98); <0.001	2.078 (1.89–2.28); <0.001
Comorbidities					
DM	1.293 (1.26–1.33); <0.001	1.166 (1.08–1.26); <0.001	1.238 (1.18–1.30); <0.001	1.133 (1.07–1.20); <0.001	1.064 (1.03–1.10); <0.001
HTN	0.850 (0.83–0.87); <0.001	0.848 (0.79–0.91); <0.001	0.842 (0.80–0.89); <0.001	0.767 (0.73–0.81); <0.001	0.849(0.82–0.88); <0.001
CKD3–5	1.002 (0.96–1.05); 0.93	1.01 (0.89–1.15); 0.871	1.273 (1.17–1.38); <0.001	1.20 (1.1–1.30); <0.001	1.425 (1.36–1.50); <0.001
ESRD	2.638 (2.49–2.80); <0.001	2.611 (2.26–3.01); <0.001	4.853 (4.47–5.28); <0.001	3.165 (2.85–3.52); <0.001	2.780(2.60–2.98); <0.001
Smoking	0.622 (0.60–0.64); <0.001	0.640 (0.59–0.70); <0.001	0.670 (0.63–0.71); <0.001	0.642 (0.60–0.68); <0.001	0.819(0.79–0.85); <0.001
Dyslipidemia	0.9 (0.88–0.92); <0.001	0.921 (0.85–1.00); 0.048	0.957 (0.91–1.01); 0.119	0.939 (0.88–0.99); 0.038	0.966 (0.93–1.00); 0.054
Overweight BMI 25 to 29.9	1.04 (0.97–1.12); 0.261	0.982 (0.79–1.21); 0.863	1.238 (1.08–1.41); 0.002	1.167 (1.01–1.35); 0.036	1.489 (1.37–1.61); <0.001
Obesity BMI 30+	0.98 (0.95–1.00); 0.165	0.954 (0.88–1.03); 0.232	1.016 (0.96–1.07); 0.55	0.964 (0.91–1.02); 0.226	0.736 (0.71–0.76); <0.001
CAD	0.98 (0.95–1.01); 0.176	0.924 (0.84–1.01); 0.099	0.999 (0.94–1.06); 0.981	0.919 (0.86–0.98); 0.014	0.986 (0.95–1.02); 0.49

**Table A3:** Detailed multivariable regression analysis for cardiac complications.

Variable	Acute Right HF Exacerbation	Cor Pulmonale	Chronic Thromboembolic PH	Cardiac Arrest
OR (95% CI); *p*-value				
IBD	0.496 (0.24–1.04); 0.065	0.324 (0.14–0.78); 0.012	0.940 (0.47–1.89); 0.862	0.647 (0.50–0.83); <0.001
Age mean (SD)	1.002 (0.99–1.01); 0.399	1.011 (1.01–1.02); <0.001	0.994 (0.99–1.00); 0.007	1.008 (1.01–1.01); <0.001
Sex (female)	0.982 (0.88–1.10); 0.741	0.933 (0.84–1.03); 0.177	1.141 (0.99–1.31); 0.064	0.852 (0.82–0.89); <0.001
Race Ref: White				
Black	1.165 (1.02–1.33); 0.027	1.023 (0.90–1.16); 0.733	1.784 (1.52–2.10); <0.001	1.503 (1.43–1.58); <0.001
Hispanic	0.762 (0.60–0.98); 0.031	0.698 (0.55–0.89); 0.003	1.342 (1.03–1.75); 0.030	1.459 (1.36–1.57); <0.001
Asian	1.210 (0.78–1.89); 0.402	0.729 (0.43–1.24); 0.242	0.916 (0.46–1.84); 0.805	1.605 (1.39–1.86); <0.001
Comorbidities				
DM	0.976 (0.86–1.11); 0.715	1.179 (1.06–1.32); 0.004	0.871 (0.74–1.03); 0.108	1.398 (1.33–1.47); <0.001
HTN	1.127 (0.99–1.28); 0.065	1.299 (1.15–1.47); <0.001	1.137 (0.96–1.34); 0.126	0.863 (0.82–0.91); <0.001
Dyslipidemia	0.809 (0.706–0.927); 0.002	0.965(0.86–1.081); 0.536	0.961 (0.809–1.141); 0.648	0.917 (0.872–0.964); <0.001
Overweight BMI 25 to 29.9	0.643 (0.42–0.98); 0.041	0.665 (0.46–0.96); 0.03	0.95 (0.60–1.5); 0.824	0.742 (0.64–0.86); <0.001
Obesity BMI 30+	1.800 (1.60–2.03); <0.001	2.346 (2.11–2.61); <0.001	1.418 (1.22–1.65); <0.001	0.961 (0.91–1.01); 0.125
CKD3–5	1.642 (1.39–1.94); <0.001	1.603 (1.39–1.85); <0.001	1.989 (1.62–2.45); <0.001	1.136 (1.06–1.22); <0.001
ESRD	1.013 (0.69–1.49); 0.948	1.300 (0.95–1.78); 0.104	0.599 (0.33–1.09); 0.095	2.874 (2.63–3.15); <0.001
Smoking	0.866 (0.76–0.987); <0.031	1.12 (1.003–1.252); 0.045	1.138 (0.972–1.333); 0.109	0.69 (0.655–0.727); <0.001
CAD	0.780 (0.67–0.92); 0.002	0.950 (0.83–1.08); 0.441	0.899 (0.74–1.10); 0.301	1.087 (1.03–1.15); 0.003
